# Exome and immune cell score analyses reveal great variation within synchronous primary colorectal cancers

**DOI:** 10.1038/s41416-019-0427-4

**Published:** 2019-03-21

**Authors:** Ulrika A. Hänninen, Erkki-Ville Wirta, Riku Katainen, Tomas Tanskanen, Jiri Hamberg, Minna Taipale, Jan Böhm, Laura Renkonen-Sinisalo, Anna Lepistö, Linda M. Forsström, Esa Pitkänen, Kimmo Palin, Toni T. Seppälä, Netta Mäkinen, Jukka-Pekka Mecklin, Lauri A. Aaltonen

**Affiliations:** 1 Applied Tumor Genomics Research Program, Research Programs Unit, University of Helsinki, Helsinki, Finland; 2 Department of Medical and Clinical Genetics, Medicum, University of Helsinki, Helsinki, Finland; 3 Department of Gastroenterology and Alimentary Tract Surgery, Tampere University Hospital, Tampere, Finland; 4 Department of Medical Biochemistry and Biophysics, Karolinska Institutet, Stockholm, Sweden; 5 Department of Pathology, Central Finland Central Hospital, Jyväskylä, Finland; 6 Department of Surgery, Helsinki University Central Hospital, Hospital District of Helsinki and Uusimaa, Helsinki, Finland; 7 Genome Biology Unit, European Molecular Biology Unit (EMBL), Heidelberg, Germany; 8 Department of Surgery, Central Finland Central Hospital, Jyväskylä, Finland; 9 Faculty of Sport and Health Sciences, University of Jyväskylä, Jyväskylä, Finland

**Keywords:** Colorectal cancer, Cancer genetics

## Abstract

**Background:**

Approximately 4% of colorectal cancer (CRC) patients have at least two simultaneous cancers in the colon. Due to the shared environment, these synchronous CRCs (SCRCs) provide a unique setting to study colorectal carcinogenesis. Understanding whether these tumours are genetically similar or distinct is essential when designing therapeutic approaches.

**Methods:**

We performed exome sequencing of 47 primary cancers and corresponding normal samples from 23 patients. Additionally, we carried out a comprehensive mutational signature analysis to assess whether tumours had undergone similar mutational processes and the first immune cell score analysis (IS) of SCRC to analyse the interplay between immune cell invasion and mutation profile in both lesions of an individual.

**Results:**

The tumour pairs shared only few mutations, favouring different mutations in known CRC genes and signalling pathways and displayed variation in their signature content. Two tumour pairs had discordant mismatch repair statuses. In majority of the pairs, IS varied between primaries. Differences were not explained by any clinicopathological variable or mutation burden.

**Conclusions:**

The study shows major diversity within SCRCs. Rather than rely on data from one tumour, our study highlights the need to evaluate both tumours of a synchronous pair for optimised targeted therapy.

## Background

Colorectal cancer (CRC) is the third most common cancer type and the fourth leading cause of cancer deaths worldwide.^1^ Around 4% of CRC patients have more than one simultaneous primary colorectal carcinoma.^2–4^ These synchronous CRCs (SCRCs) are more common in men as CRC in general, but the male–female ratio is even higher than with solitary tumours.^3,4^ Compared to solitary CRCs, clinicopathological studies have associated SCRC more often with mucinous histology, right-sided tumours, presence of adenomas, and sessile serrated adenomas as the precursor.^5^ There is no consensus on the correlation between the age at diagnosis and SCRC. Studies tend to support a higher age at presentation in SCRC than with solitary tumours.^6,7^ SCRC prognosis compared to that of solitary tumours vary between studies with contradicting results.^2,4,5,7,8^ The majority of CRCs are microsatellite stable (MSS), whereas about 15% of CRCs display microsatellite instability (MSI).^9^ MSI tumours have a defective DNA mismatch repair (MMR) system and thus exhibit a remarkably high mutation burden compared to MSS tumours. Some studies have shown that MSI tumours are more frequent in SCRC than in solitary colorectal carcinomas,^5,8^ but this may be due to underlying Lynch syndrome (LS).

SCRC has been associated with predisposing conditions, such as LS, familial adenomatous polyposis, and inflammatory bowel disease.^6,10–12^ According to a large-scale study, such conditions account for only slightly more than 10% of SCRC cases.^3^ The pathological and clinical features of the SCRCs have been widely studied.^2,3,5^ The underlying molecular mechanisms in SCRC, however, have not been thoroughly examined. Genetic research has mainly focused on single factors such as MSI status and known cancer genes, including *APC*, *KRAS*, and *BRAF*, with varying results.^8,13–15^ Thus far, there has been only a few exome-wide studies on SCRC and one study including whole-genome sequencing data of a SCRC case.^16–19^ In general, these studies have supported the idea of independent origins of synchronous tumours. Another hypothesis underlying SCRC, in addition to a common progenitor, is the field effect.^8,20^ The molecular basis for the field effect has been proposed to be e.g. massive exposure to a carcinogen or genetic predisposition through mosaicism in a patient. Epigenetic alterations, such as low LINE-1 methylation, O^6^-methylguanine-DNA methyltransferase promoter methylation, or other CpG island methylation in the colonic mucosa, and damaging germline mutations in immune-related genes leading to an inflammatory microenvironment that might favour tumorigenesis, have also been suggested as potential causative mechanisms.^16,21–23^

By exome sequencing the tumour pair and corresponding normal sample from 23 patients, the largest exome set of SCRCs thus far, we studied comprehensively the extent of genetic overlap within the synchronous tumours, including mutational signatures, and searched for possible germline predisposing genes. In addition, we evaluated the lymphocyte levels (immune cell score, IS) in the tumours using the same principle as Immunoscore^®^, which derives from the densities of CD3+ and CD8+ lymphocytes in the tumour centre and invasive margins, a new prognostic marker in CRC. Immune score combined with TNM classification and/or defined MMR status has been suggested to predict survival and response to therapy in colon cancer.^24–27^ It is not known how immune responses and thus immune cell levels vary between synchronous tumours within a patient. Knowledge on the molecular basis of SCRC provides more insight on how these tumours arise and allows the development of more personalised treatments.

## Materials and methods

### Patient cohort

The study set composed of 23 CRC pairs and their corresponding normal tissue samples. All patients were Finnish (white Caucasian) of which 20 patients were derived from a consecutive, population-based series of 1088 CRCs (Suolisyöpä Keski-Suomi) from Jyväskylä, Finland. In addition, 3 SCRC patients were derived from an in-house population-based series of 1042 CRCs collected 1994–1998^28,29^ and a subsequent, ongoing series of CRCs collected since 1998 (unpublished data). We included only SCRC patients from which we had material from both tumours. For two of the in-house cases, we utilised readily available whole-genome sequencing data for one tumour and normal sample, while exome sequencing the other tumour. All relevant medical records were available for all the cases.

Some previous studies have considered lesions occurring within 6 months of the primary excisional operation as synchronous. Here we defined the term synchronous by having at least two tumours diagnosed and operated simultaneously. These tumours were all separated by normal colonic mucosa and defined as independent primary tumours both by a surgeon and a pathologist. All except one patient (sync_11 who belongs to a LS family with a pathogenic *MLH1* germline variant) were assumed sporadic. None of the patients had a known history of inflammatory bowel disease. Four patients had three simultaneous cancers. From one of them (sync_11) all three tumours were included in this study.

Samples were mainly formalin-fixed and paraffin-embedded (FFPE) with a few fresh frozen tissue samples (59 FFPE and 11 fresh frozen). MMR status was determined by immunohistochemistry staining (Suolisyöpä Keski-Suomi samples) and MSI status with microsatellite markers (in-house samples). The division of MSS and MSI tumours was confirmed by the exome data (see Supplementary Table 1).

### DNA extraction

For genomic DNA extraction from FFPE blocks, we used a standard phenol-chloroform isolation method, and from fresh frozen tissue, non-enzymatic DNA extraction protocol. DNA concentration was determined with the Qubit double-stranded DNA BR Assay Kit (Thermo Fisher Scientific, Waltham, MA, USA) and purity with NanoDrop 8000 (Thermo Fisher Scientific).

### Exome sequencing

Exome libraries were prepared with KAPA Hyper Prep Kit (Kapa Biosystems, Wilmington, MA, USA). Coding exons and untranslated regions of the genome (94 megabases) were enriched with the NimbleGen SeqCap EZ Exome Library v3 Kit (Roche NimbleGen, Madison, WI, USA). Paired-end sequencing with read lengths of 75 base pairs with a median depth of 37× (interquartile range (IQR), 33× to 40×) was performed by Illumina HiSeq 2000/4000 (Illumina Inc., San Diego, CA, USA) in Karolinska Institutet, Sweden. The median fraction of the exome with at least 10× coverage was 84%.

### Read mapping and variant calling

The quality of raw sequencing data was examined using FastQC v.0.10.0 (http://www.bioinformatics.bbsrc.ac.uk/projects/fastqc/) and QualiMap v.2.1 (http://qualimap.bioinfo.cipf.es/).^30^ 3′ ends of reads with high adapter similarity were removed with Trim Galore! v.0.3.07 (http://www.bioinformatics.babraham.ac.uk/projects/trim_galore/) and trimmed reads were mapped to the integrated 1000 Genomes Phase 2 GRCh37/hg19 reference assembly with Burrows-Wheeler Aligner (BWA)–MEM version 0.7.12 (http://bio-bwa.sourceforge.net/).^31^ Overlapping read pairs were clipped using BamUtil version 1.0.13 (http://genome.sph.umich.edu/wiki/BamUtil#Releases) ClipOverlap. Duplicate reads were removed using Samtools v.1.0 (http://www.htslib.org/)^32^ rmdup on both paired-end and single-end reads to correct for e.g. FFPE-derived sequencing artefacts. Aligned reads were locally realigned using the Genome Analysis ToolKit (GATK) v.3.5 (https://www.broadinstitute.org/gatk/) IndelRealigner^33^ and base scores were recalculated with GATK BaseRecalibrator. After realignment the final single-nucleotide variant (SNV) and indel calls were made with the GATK HaplotypeCaller using a variant quality threshold of 1.0.

### Somatic variant analysis

The tumour data were filtered against the normal tissue data to remove germline variants. We refined remaining variant calls against a pooled set of whole-genome sequencing data (median ~40× coverage/sample) from 10 blood samples by excluding any SNV call, which was found in three or more reads in the pooled data. Indel calls were filtered out if two or more samples had more than three reads calling an indel at 100 base pairs (read length) from the indel locus. This step was done to exclude low allelic fraction artefacts in regions prone to sequencing errors. Only variants within the targeted region of NimbleGen SeqCap EZ Exome Library v3 Kit were analysed.

A comparative analysis and visualisation tool BasePlayer^34^ was utilised to visualise and analyse the data. The criteria to call a mutation included total coverage of at least four reads at the mutation locus with the GATK quality score at least 20, to filter out false calls that derive e.g. from FFPE. For indels, the criteria required additionally a minimum of 10% mutant allele fraction. Indels called with the minimum mutant allele fraction (*n* = 31) had a total coverage of 50 or higher. In addition, 1000 Genomes phase 1 pilot-style callability region mask was used to filter out possible false calls located in regions with poor callability.^35^ The comparison of the mutation profiles to determine the amount of genetic overlap in each tumour pair took into account the number of shared variants that occurred in the exact same positions in the coding region.

### Germline variant analysis

The normal tissue data were filtered against The Genome Aggregation Database (gnomAD, http://gnomad.broadinstitute.org/).^36^ Germline variants with allele frequency more than 0.001 in the whole gnomAD, 0.001 in population-specific Finnish gnomAD exome set (*n* = 85,202), and 0.01 in population-specific Finnish genome set (*n* = 1747) were excluded. Indel calls were refined similarly as in the somatic variant analysis. The criteria to call a variant included coverage of at least 10 reads, GATK quality score of at least 20, and a minimum of 20% mutant allele fraction. Only coding variants within the targeted region of NimbleGen SeqCap EZ Exome Library v3 Kit were analysed.

### Mutation signature analysis

Mutational signatures were modelled in 96-dimensional space representing all single base mutations in one base pair context, possibly reversely complemented such that the mutation source base is always cytosine or thymine. Each tumour is represented by an integer vector of counts of such somatic mutations. The mutation count in a given sequence context was modelled as Poisson distributed random variable with the rate parameter being a linear combination of four distinct Dirichlet(1) distributed mutational signatures: one specific to patient, one related to MSI, and two tumour-specific processes. The prior for the signature weights was half Cauchy(100). The modelling and Markov chain Monte Carlo (MCMC) sampling was performed with pymc3 library.^37^ We drew 10,000 MCMC samples from 10 independent chains ignoring 500 burn-in samples and thinning with factor 100. Signature comparison to Alexandrov et al. was performed using Maximum-A-Posteriori probability signatures.^38^ To calculate the variation of signature contents between tumours mixed linear model “Y = m + MMRstatus + Individual + Tumour” was used on MAP estimates of signature exposure proportion Y, explained by fixed effects of intercept m and MMR status and random effects of Individual and Tumour.

### Ingenuity pathway analysis

Ingenuity pathway analysis (IPA) version 44691306 was used to determine the frequency of known cancer pathways affected within the tumour pairs. IPA was utilised to define genes linked to each pathway. All genes with at least one non-synonymous mutation were included in the analysis.

### Sanger sequencing

The reported mutations in *XPNPEP1*, *FAM133A*, and *GPR98* were all validated successfully with Sanger sequencing (recall rate 100%). The corresponding normal samples were also sequenced to confirm somatic nature of mutations. Primers were designed using Primer3Plus.^39^ Regarding FFPE tissue samples, three independent PCR reactions were performed to ensure consistency of the observations. Sequencing reactions were performed with the Big Dye Terminator v.3.1 kit (Applied Biosystems, Foster City, CA, USA) on an ABI3730 Automatic DNA Sequencer (FIMM Technology Center and DNA sequencing and Genomics laboratory, Institute of Biotechnology, Helsinki, Finland). The sequence graphs were analysed both with the Mutation Surveyor –software (version v4.0.8, Softgenetics, State College, PA) and manually.

### Immune cell score determination

Immunohistochemical staining with CD3 (Novocastra, NCL-L-CD3, clone PS1) and CD8 (Thermo Scientific, RM-9116, clone SP16) antibodies was performed as previously described.^26^ Stained whole-section slides were digitally scanned with NanoZoomer-XR (Hamamatsu Photonics K.K., Hamamatsu City, Japan), and positively stained T-cell counts were calculated using QuPath image analysis software.^40^

The IS consists of CD3 and CD8 lymphocyte counts from tumour centre and invasive margin. Width of the invasive margin was considered to span 360 µm into the tumour and 360 µm into the healthy tissue from the visible tissue frontier as presented by Hermitte.^41^ This area was marked with an annotation brush tool. A representative area within the tumour centre was selected for cell calculation. From both CD3 and CD8 stainings mean analysed area was 10.0 mm² for invasion margin and 13.6 mm^2^ for tumour centre. The sections were determined to have either high or low lymphocyte count (number of cells/mm^2^). To form IS we used the same receiver-operating characteristic (ROC) curve-based cut-off values that were used in a previous study with a larger study population.^26^ Areas with low lymphocyte count were scored as 0 and areas with high lymphocyte count were scored as 1, so the following IS gained a value from 0 to 4. IS is composed using the same basic principles as Immunoscore^®^ with the difference of manually selected representative tumour areas and invasive margins, ROC curve-based cut-off values, and the use of open source image analysis software.

### Statistical analysis of IS data

We utilised R version 3.5.1 to perform statistical analyses. Spearman’s rank correlation coefficient was used to evaluate the correlation of IS within tumour pairs, excluding one case with three tumours (sync_11). Considering that tumours from the same individual may not be independent, we applied generalised estimating equations (GEEs) with ordinal response to examine the association between IS and clinicopathological variables.^42^ The ordLORgee function from the multgee package was used to fit GEE models with uniform local odds ratios and cumulative logit link.^43^ *P*-value < 0.05 was regarded as statistically significant. All reported *P*-values are two-sided.

## Results

### Cohort characteristics

We analysed a total of 47 tumours from 23 patients. Table 1 presents the clinicopathologic features of these cases. The majority of patients (18/23, 78%) were male. The median age at surgery was 72 years (range, 48–83 years). Majority of the tumours (31/47, 66%) were located on the left side of the colorectum (splenic flexure, descending or sigmoid colon, or rectum), whereas 16/47 (34%) were right-sided (caecum, ascending, or proximal transverse colon). Altogether 18/23 (78%) tumour pairs resided on the same side, the majority (13/18, 72%) being left-sided. Eighteen tumour pairs were MSS-MSS, three MSI-MSI, and two MSS-MSI. Four patients had an additional tumour (of which one was included in the study) in the colorectum and 18 patients presented with at least one synchronous adenoma.

**Table 1:** Cohort characteristics

Patient	Sex	Age	Tumour	Location	Histology	Grade	MSI	TNM	T size (cm)	Adenomas
s894	M	79	s894–1	Descendens	ac	2	MSS	T2N0M0	6	Yes
			s894–2	Descendens	ac in situ	2	MSS	Tis	1.2	
c440	M	83	c440–1	Border of rectosigma	ac in situ	1	MSS	Tis	NA	Yes
			c440–2	Caecum	ac	3	MSI	T3N0M0	4.5	
sync_1	M	82	sync_1–1	Caecum	ac	3	MSS	T2N0M0	3.5	No
			sync_1–2	Transversum	ac	3	MSS	T3N0M0	5	
sync_2	M	82	sync_2–1	Rectum	ac	2	MSS	T4N0M1	4.3	Yes
			sync_2–2	Rectum	ac in situ	1	MSS	Tis	NA	
sync_3	F	67	sync_3–1	Rectum	ac	2	MSS	T3N1M0	NA	Yes
			sync_3–2	Rectum	ac	2	MSS	T1	NA	
s75	M	71	s75–1	Rectum	ac muc	3	MSS	T3N0M0	4	Yes
			s75–2	Caecum	ac	2	MSS	T3N0M0	5	
s1268	M	66	s1268–1	Ascendens	MANEC	4	MSS	T3N2M0	5.2	Yes
			s1268–2	Sigma	ac	2	MSS	T3N1M1	4	
s882	F	67	s882–1	Rectum	ac	2	MSS	T1	1	No
			s882–2	Caecum	ac	2	MSS	T4N0M0	2	
sync_4	M	78	sync_4–1	Ascendens	ac muc	3	MSI	T3N0M0	11	Yes
			sync_4–2	Ascendens	ac muc	3	MSI	T3N0M0	8	
s387	M	62	s387–1	Flexura lienalis	ac	1	MSS	T3N1M1	3.5	NA
			s387–2	Flexura lienalis	ac	1	MSS	T3N1M1	8	
sync_6	M	72	sync_6–1	Caecum	ac	2	MSS	T4N1M0	4.1	Yes
			sync_6–2	Ascendens	ac	2	MSS	T2N0M0	4.7	
s483	F	81	s483–1	Caecum	ac muc	3	MSI	T3N2M0	9	Yes
			s483–2	Ascendens	ac	1	MSI	T1	1.5	
s956	M	76	s956–1	Rectum	ac	2	MSS	T3N1M0	6	Yes
			s956–2	Sigma	ac	2	MSS	T3N1M0	4	
s404	M	61	s404–1	Sigma	ac	2	MSS	T1N1M0	3.4	Yes
			s404–2	Sigma	ac	2	MSS	T2N1M0	5.5	
sync_7	M	72	sync_7–1	Rectum	ac	2	MSS	T2N0M0	4	Yes
			sync_7–2	Sigma	ac	2	MSS	T1	4	
sync_9	M	80	sync_9–1	Rectum	ac	2	MSS	T2N0M0	3	Yes
			sync_9–2	Sigma	ac	2	MSS	T1	NA	
sync_10	M	59	sync_10–1	Rectum	ac	2	MSS	T3N1M0	4	No
			sync_10–2	Sigma	ac	2	MSS	T4N1M0	4.5	
sync_11	M	48	sync_11–1	Ascendens	ac	2	MSI	T3N0M0	3.8	Yes
			sync_11–2	Ascendens	ac	2	MSI	T3N0M0	11	
			sync_11–3	Ascendens	ac	2	MSI	T1N0M0	1	
s1036	M	77	s1036–1	Sigma	ac	2	MSS	T4N0M0	12	Yes
			s1036–2	Sigma	ac	2	MSS	T1N0M0	1.7	
s1283	F	75	s1283–1	Caecum	ac	2	MSI	T3N0M0	3.5	Yes
			s1283–2	Sigma	ac	NA	MSS	T4N2M0	2.5	
c110	M	49	c110–1	Rectum	ac	2	MSS	T3N0M0	5	Yes
			c110–2	Descendens	ac	2	MSS	T3N0M0	3.2	
c117	F	83	c117–1	Descendens	ac	2	MSS	T3N1M0	7.5	Yes
			c117–2	Rectum	ac	2	MSS	T3N0M0	5	
s934	M	51	s934–1	Rectum	ac	2	MSS	T2N0M0	4	No
			s934–2	Sigma	ac	2	MSS	T3N2M0	5	

*MSI* microsatellite unstable, *MSS* microsatellite stable, *ac* adenocarcinoma, *MANEC* mixed adenoneuroendocrine carcinoma, *ac muc* adenocarcinoma mucinosum, *NA* not available

### Mutation spectrum

Exome sequencing analysis identified 18,134 somatic coding mutations across all samples. The most common transition type in the tumours was C>T that accounted for 60% of all the detected mutations. The median number of non-synonymous mutations per sample was 88.5 in MSS tumours (IQR, 60.3–120) and 933 in MSI tumours (IQR, 637–1197). There was variation in the number of non-synonymous mutations within tumour pairs. The median difference per MSS-MSS tumour pair was 44 non-synonymous mutations (IQR, 15.5–71.8). The mutation count within the three MSI-MSI pairs differed by 193, 359, and 902 mutations (sync_11 with three tumours having largest difference between lowest and highest).

### Lack of genetic overlap within tumour pairs points to separate origins

**Fig. 1:**
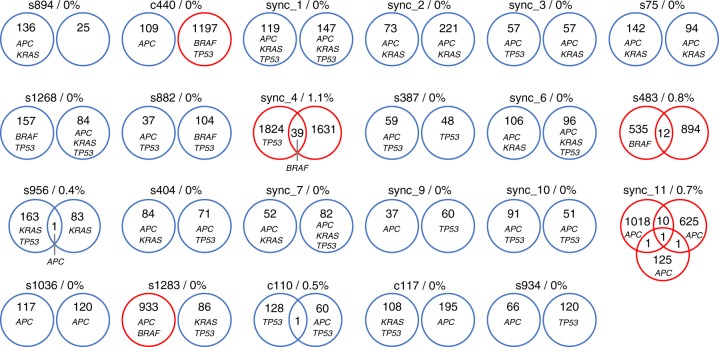
Number of shared non-synonymous mutations in tumour pairs. Nearly all mutations were unique within paired tumours. Mutated *APC*, *KRAS*, *BRAF*, and *TP53* are marked in the tumours. There were only two cases where the paired tumours shared an exact same change in one of these genes. Blue = microsatellite stable tumour; red = microsatellite unstable tumour

The majority of the mutations were unique within tumour pairs (Fig. 1). Out of 23 pairs, only 5 (22%) shared at least one exact same mutation within paired lesions (Supplementary Table 2). In three of these pairs the tumours shared the same driver mutation either in *BRAF*, *APC*, or *ACVR2A* (Fig. 1). Altogether, shared mutations accounted for 0.4–1.1% of the mutations found within the five pairs. Of these pairs, two were MSS-MSS that shared one mutation per pair: s956 in *APC* (R213X) and c110 in *GPR98* (S4503C). The other three pairs were MSI-MSI that shared 12–39 mutations. All except two of these shared variants (*BRAF* hotspot V600E in sync_4 and *XPNPEP1* R204H in sync_11 (tumours sync_11–2 and sync_11–3)) were frameshift mutations. One of these frameshift mutations, shared by two sync 11 tumours, was in *RPL22*, a gene that was recently reported to be frequently mutated in SCRC.^18^ This mutation was found altogether in 5 out of 47 tumours (11%), all of which were MSI.

Next, we investigated whether tumours within a pair would harbour mutations in the same known CRC genes. Discordance in the mutation statuses was seen in many of these genes (Table 2, Supplementary Table 3). Six out of 23 (26%) patients had a tumour pair with one *KRAS* mutant and one wild type tumour. *BRAF*, another clinically relevant gene, was mutated in one tumour of a pair in 5/23 (22%) cases. This discordance was also evident in other genes with possible clinical relevance, such as *PIK3CA* in 6/23 (26%) and *PTEN* in 3/23 (13%). There were no known cancer genes whose mutation patterns (mutated in both or only one tumour of a patient) would have correlated across all pairs.

**Table 2:** Mutations in known cancer genes

Among the most frequently mutated genes in the whole sample set were the known cancer genes *APC* (33/47, 70%), *TP53* (23/47, 49%), *KRAS* (18/47, 38%), *TCF7L2* (11/47, 23%), and *ACVR2A* (10/47, 21%). Exact same changes that occurred within four tumour pairs are marked with a hashtag. Sample names marked with red refer to MSI tumours. Tumours might also contain additional mutations in these genes

As expected, differences existed between MSS and MSI tumours. Among the most frequently mutated genes in MSS tumours were the known cancer genes *APC* (29/38, 76%), *TP53* (21/38, 55%), *KRAS* (18/38, 47%), and *TCF7L2* (7/38, 18%). In MSI tumours, the most frequently mutated known cancer genes were *ACVR2A* (9/9, 100%), *MSH3* (5/9, 56%), *FBXW7* (5/9, 56%), and *BRAF* (5/9, 56%). CRC genes that were affected only in MSS tumours were *KRAS* and *NRAS*, whereas genes exclusive to MSI tumours included *MLH1*, *MLH3*, *MSH2*, *MSH3*, and *ERBB2*.

Finally, we searched whether tumours within the pairs would have other mutated genes in common. We focused on genes with a truncating mutation in both tumours. Excluding the exact same mutations and the known cancer genes listed above, there were altogether four tumour pairs with such genes (Supplementary Table 4). One MSI-MSS (s1283) pair displayed mutations in *FAM133A*, whereas three MSI-MSI pairs shared each 3–34 genes with truncating mutations, mainly consisting of frameshift mutations.

### Frequently mutated signalling pathways

Even though paired tumours harboured mutations in different genes, we examined if these genes fell into similar pathways. We characterised the most frequently mutated known cancer pathways (ATM, p53, Wnt/β-catenin, ERBB, PTEN, PI3K/AKT, ERK/MAPK, and TGF-β) in tumour pairs (Supplementary Table 5). The number of overlapping altered known pathways varied between one and eight in a pair. There were six pairs where all the eight pathways studied were mutated in both tumours. However, the number of mutated genes associated with the pathways varied within the tumour pairs.

### Mutational signatures vary within tumour pairs

Next, we performed Bayesian mutation signature analysis for the whole tumour set to see whether the tumours had experienced different mutational processes. The mutation count in a given sequence context was considered Poisson distributed with the rate parameter being a linear combination of four distinct Dirichlet distributed mutational signatures: one specific to patient, one related to MSI, and two signatures, ts1 and ts2, that were tumour-specific. The median number of mutations attributable to each signature was 120, 1 581, 56.9, and 16.2, respectively. The patient-specific signature was similar to the known age-related signature 1, reported by Alexandrov et al.^44^ The paired tumours harboured similar numbers of mutations contributing to patient-specific signature, which suggests that tumours within a patient were of similar molecular age. The MSI signature was similar to signature 6, the known MSI signature. The two tumour-specific signatures did not clearly correspond to any of the known signatures.

For MSS tumours, 44–53% (95% credible interval (CI)) of mutations could be attributed to tumour-specific mutational processes in contrast to the processes intrinsic to the patient. For MSI tumours, the tumour-specific proportion of somatic mutations not attributed to MMR defect was probably (94.7% posterior probability) lower, 27–51%, reflecting the decreased demand for oncogenic mutations in the tumours beyond the ones produced by MSI. Non-MSI-related tumour-specific signatures accounted for 85–71% fewer mutations in MSI tumours as they did in MSS tumours. The MMR defect itself produced 5–8 times (95% CI) the number of mutations that the patient-intrinsic and tumour-specific, non-MSI mutation processes did.

To assess whether tumours within a patient resemble each other more than other tumours in the set in general, the proportion of variation of both tumour-specific signatures within pairs were compared to the variation of these signatures in the whole set. Patient identity explained approximately 35% of the variance in relative contribution of ts1 mutation signature while ts2 was entirely independent of the patient. Much of the variance contribution of ts1 is explained by its association with freshly frozen instead of formalin-fixed samples. After accounting for the sample processing differences, only 8% of individual attributable variance remains.

Looking at signature fractions, within 11/18 (61%) MSS-MSS pairs, the order of most contributing signatures was the same (Supplementary Figure 1). Of these, 9 pairs had the individual signature as the biggest contributor, followed by ts1. In 2 pairs the ts1 contributed the most and individual signature second. Within 7 MSS-MSS pairs the tumours displayed different order of signature fractions. Location of the tumours did not explain the variation in signature fractions within pairs. The majority (6/7, 86%) of the tumour pairs with different signature order located in the same side.

### Absence of common predisposing factors in SCRC

We analysed the germline variants of the 23 SCRC patients to search for candidate susceptibility genes. The one known Lynch syndrome patient (sync_11) had a germline mutation in *MLH1* (rs193922370, c.454–1G>A). No other known predisposing factors were found. Patient sync_4 harboured a missense variant in *APC* L387V (rs760312726) in the germline. This variant was found in five Finnish controls in gnomAD (minor allele frequency = 0.0002245). Careful examination of the patient records did not reveal signs of familial adenomatous polyposis. When looking at shared variants, we detected no clear candidate predisposing genes in this sample set.

### Immune cell counts vary between lesions

IS was assessed in 39 tumours from 19 SCRC patients (Supplementary Table 6). The highest immunoscore category (IS4) was observed most frequently (IS0, 15%; IS1, 10%; IS2, 15%; IS3, 18%; and IS4, 41%). Equal immunoscores were observed in 3 patients (3/19, 16%), whereas IS varied by one point in 6 patients (6/19, 32%), and by at least two points in 10 patients (10/19, 53%). Paired tumours did not show a significant correlation in IS (*P* = 0.837).

Immune response was generally stronger in MSI tumours compared to MSS tumours (78% MSI tumours with IS3 or IS4 vs. 53% MSS tumours with IS3 or IS4), although the difference was not statistically significant (*P* = 0.103). Mutation count (*P* = 0.441), gender (*P* = 0.397), or age at diagnosis (*P* = 0.339) did not seem to explain the differences in IS. Also, there were no clear differences in IS between left- and right-sided tumours (*P* = 0.161). Of note, all the MSI tumours were right-sided and most of the MSS tumours (77%) were left-sided. IS was not associated with mutation signatures (individual signature, *P* = 0.288; signature ts1, *P* = 0.151; and signature ts2, *P* = 0.463).

**Fig. 2:**
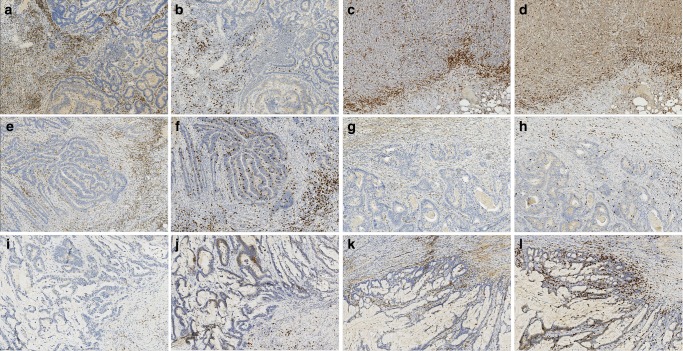
Examples from CD3 and CD8 stainings showing part of tumour and invasive margins from synchronous microsatellite stable - microsatellite unstable (MSS-MSI), MSS-MSS and MSI-MSI tumour pairs. **a**–**d** MSS-MSI tumour pair: MSS c440-1 with IS3 and MSI c440-2 with IS4 (**a** and **b** are CD3 and CD8 stainings from c440-1 and **c** and **d** CD3 and CD8 stainings from c440-2, respectively). **e**–**h** MSS-MSS tumour pair: s404-1 with IS4 and s404-2 with IS1 (**e** and **f** are CD3 and CD8 stainings from s404-1 and **g** and **h** CD3 and CD8 stainings from s404-2, respectively). **i**–**l** MSI-MSI tumour pair: sync 4-1 with IS0 and sync 4-2 with IS4 (**i** and **j** are CD3 and CD8 stainings from sync 4-1 and **k** and **l** CD3 and CD8 stainings from sync 4-2, respectively). Photos were captured with a ×20 magnification

Out of four pairs with the largest difference, three pairs resided in the same segment (one pair on the right and two pairs on the left side). Mutation statuses of the known cancer genes or clinicopathological variables did not explain the IS variation within patients. As an example, the tumours of a pair with the largest IS difference (IS0 and IS4) were both MSI, located in ascending colon, mucinous, and stage T3 tumours (Fig. 2). Similarly, within two patients harbouring a MSS-MSI tumour pair with considerable clinicopathological differences, IS differed maximally only by one point. One pair was grade 1 MSS Tis (tumour in situ) with a low number of mutations and IS3 from the recto-sigmoidal border and grade 3 MSI T3 tumour with a high mutation load and IS4 from caecum. The other pair was a MSS T4 tumour with a low number of mutations and IS3 from sigmoid (grade unknown) and a grade 2 MSI T3 tumour with a high mutation load and IS3 from caecum (Fig. 2). Also, when focusing on MSS-MSS pairs, variation was present. There were two patients with left-sided MSS tumours and similar mutation counts, but the IS differed by three points within the pairs (Fig. 2).

## Discussion

To the best of our knowledge, this is the largest study to date to use exome-wide genomic profiling combined with IS analysis to determine similarities and differences within SCRC pairs. Based on our exome data, only 5 out of 23 tumour pairs displayed one or more exact same genomic changes within them. Of these, 3 were MSI-MSI pairs where the tumours shared mutations almost exclusively in repeated sequences and the remaining few in known mutation hotspots. The two MSS-MSS tumour pairs both shared only one variant. One of these was a mutation hotspot in *APC*, a well-known driver gene of intestinal tumorigenesis, shared by coincidence or convergent evolution. Different mutation patterns with few shared mutations strongly suggest these tumours to have independent origins, supporting the theory of a stochastic process in the genesis of SCRC. Similar interpretation has been proposed in previous exome-level studies,^16–18^ whereas one recent single-nucleotide polymorphism array-based study suggested a subset of SCRCs to be monoclonal.^45^

Further evidence that SCRCs are independent in origin came from the observed differences in the mutation statuses of known CRC genes within tumour pairs, the phenomenon also seen in previous studies.^15–18^ Additionally, the search for other genes with a possibly damaging truncating mutation in both tumours of a pair discovered mainly genes with frameshift mutations in repeated sequences of MSI-MSI tumours, which probably have arisen by chance due to the replication errors characteristic of these type of tumours. Our data also suggest that SCRCs within patients may utilise different signalling pathways. Genomic diversity was evident in all tumour pairs, regardless whether the patient had a known predisposition syndrome, a varying number of adenomas, proximal or distal location of the tumours, or their distance from each other. Thus, our results are not in accordance with a previous study showing that similarities of molecular genetic changes within SCRC pairs are related to proximity.^14^

Recently, *RPL22* hotspot mutations were reported as frequent events in SCRCs and proposed as a potential biomarker.^18^ These mutations were present almost exclusively in MSI tumours. In this study, *RPL22* was mutated in 11% of tumours, all of which were MSI being a frequent event in MSI tumours (5/9, 56%). Since *RPL22* is a known MSI target gene and the hotspot in question is found to be mutated significantly in MSI CRC,^46–48^ we hypothesise that this phenomenon might be first and foremost related to MSI as such, rather than SCRCs.

Altogether, 22% of SCRC patients had at least one MSI tumour. The fraction of MSI tumours has been reported to be higher in SCRC than that of solitary tumours (~15%), which our results also support.^8,9,49^ The mutation frequencies of known CRC genes in SCRCs resembled those seen in solitary tumours.^50^ Even genes whose mutation frequencies have been reported to differ in SCRC, such as *BRAF*, *SMAD4*, *PIK3CA*, and *NRAS*, showed no clear difference in our study.^8,17^

Even though the SCRCs do not seem to share an origin, we evaluated whether tumour pairs would have developed through similar mutational processes by performing mutation signature analysis. Paired tumours resembled each other somewhat more than other tumours in the set when looking at tumour-specific signature ts1, whereas ts2 did not differentiate between tumours from same or different patients. Overall, signature contents resembled each other within majority of the MSS-MSS tumour pairs and also between patients. Over one-third of the tumour pairs, however, harboured different mutation signature compositions within a patient indicating these tumours have probably undergone different mutational processes.

By evaluating IS, we were able to study the immune infiltrate in both tumours of a patient to further characterise the differences and similarities. Lymphocyte count has been shown to have a prognostic value.^26,27^ The intratumour immune reaction varied between patients and, in the majority of the cases (16/19, 84%), also within tumour pairs. Variation was not clearly explained by any clinicopathologic feature, such as MSI status, mutation count, or tumour location. It is thought that the immune reaction is affected by several factors, such as the genetic background of the patient and the gut microbiota.^27^ In addition, due to the intrapersonal heterogeneity within the tumour immune reactions, our results support the notion that also more local and tumour-specific reasons can exist, such as the tumour microenvironment and the genetic content of the tumour. For example, in addition to T cells other immune cells are known to contribute to the behaviour of cancer cells.^51^

The study set consisted of solely Finnish patients, who as a population have a unique genetic makeup due to strong genetic isolation and relative small number of founder individuals, but in addition to *MLH1* variant in the LS case, no clear candidate predisposition genes were discovered. We acknowledge that to be successful this approach might require a larger sample size. The study material included DNA derived from FFPE samples. We have taken steps to ensure the efficient removal of possible false calls caused by FFPE, however, we recognise that the data may still contain some low-frequency artefacts. As a limitation of our study, we were not able to examine the epigenetic changes, such as gene methylation, or the non-coding genomic regions. Thus, it remains unknown whether these factors could have an impact on the tumour multiplicity. Another theorised factor thought to contribute to synchronous tumorigenesis is the field effect. The majority of the tumour pairs had a tendency to colonise the same side of the colorectum, which might imply possible regional field effect. Additionally, several patients had at least one additional adenoma, which could also suggest a potential field effect.

The variation within SCRC pairs may impact genetic testing and therapeutic strategies. Our results strengthen the notion that not only do the tumour pairs show variation in common drug targets or predictors of therapy response, such as *BRAF*, *KRAS*, and *PIK3CA*,^13,52^ but also in other potential targets or genetic variants that might have an effect on treatment response.^17,18^ There were many tumour pairs where one tumour displayed *KRAS* wild type and the other *KRAS* mutant status, a setting in which only one of the tumours might respond to anti-epidermal growth factor receptor therapy.^53^ Some patients displayed a combination of MSS-MSI tumours where neoantigen levels might vary between lesions. This might be relevant when considering immune checkpoint inhibitors as potential treatment options.^54^ Incorporating IS as a measure of immune response might provide further information on which tumours might react to immune modulating therapy. Thus, therapeutic decisions especially regarding targeted therapies require careful planning and it is paramount that all lesions within a patient should be analysed for optimal outcome.

In conclusion, our study proposes a parallel evolution of SCRC pairs without a common origin. This confirms previous observations and highlights the necessity to examine both tumours when designing treatment. Our study elucidates further the genetic background of SCRC and how immune responses vary between the tumours, improving our understanding of the diversity within the tumour pairs. Additional studies are still needed, however, to shed further light on the causes for tumour multiplicity.

## Supplementary information

Supplementary Figure 1. Mutational signatures in each tumor pair

Supplementary Table 1. Mutation statistics from exome sequencing data

Supplementary Table 2. Exact same changes within each pair

Supplementary Table 3. Mutations in known cancer genes

Supplementary Table 4. List of genes with two different truncating mutations within a pair

Supplementary Table 5. The altered known cancer pathways in synchronous cancers

Supplementary Table 6. IS measurements of 19 SCRC pairs

## Data Availability

Somatic sequence data generated and analysed during the current study have been deposited at the European Genome-phenome Archive (EGA), which is hosted by the EBI and the CRG, under study accession number (EGAS00001003474). Further information about EGA can be found on https://ega-archive.org “The European Genome-phenome Archive of human data consented for biomedical research” (http://www.nature.com/ng/journal/v47/n7/full/ng.3312.html). Data are available on request upon publication from the EGA database by contacting the data access committee (DAC accession EGAC00001000649, dac-tumorgenomics@helsinki.fi) assigned for this project. Data are restricted due to reasons of patient confidentiality.
