# Supplementary material for: Exome and immune cell score analyses reveal great variation within synchronous primary colorectal cancers
Source: Br J Cancer. 2019 Mar 21;120(9):922–30. doi: 10.1038/s41416-019-0427-4 (PMC6734647; doi:10.1038/s41416-019-0427-4)
Supplement: Supplementary file 1 — Supplementary Figure 1. Mutational signatures in each tumor pair [file 41416_2019_427_MOESM1_ESM.pdf]

**Supplementary Figure 1. Mutational signatures in each tumor pair.** The figure presents fractions of each signature explaining the mutations within tumors.

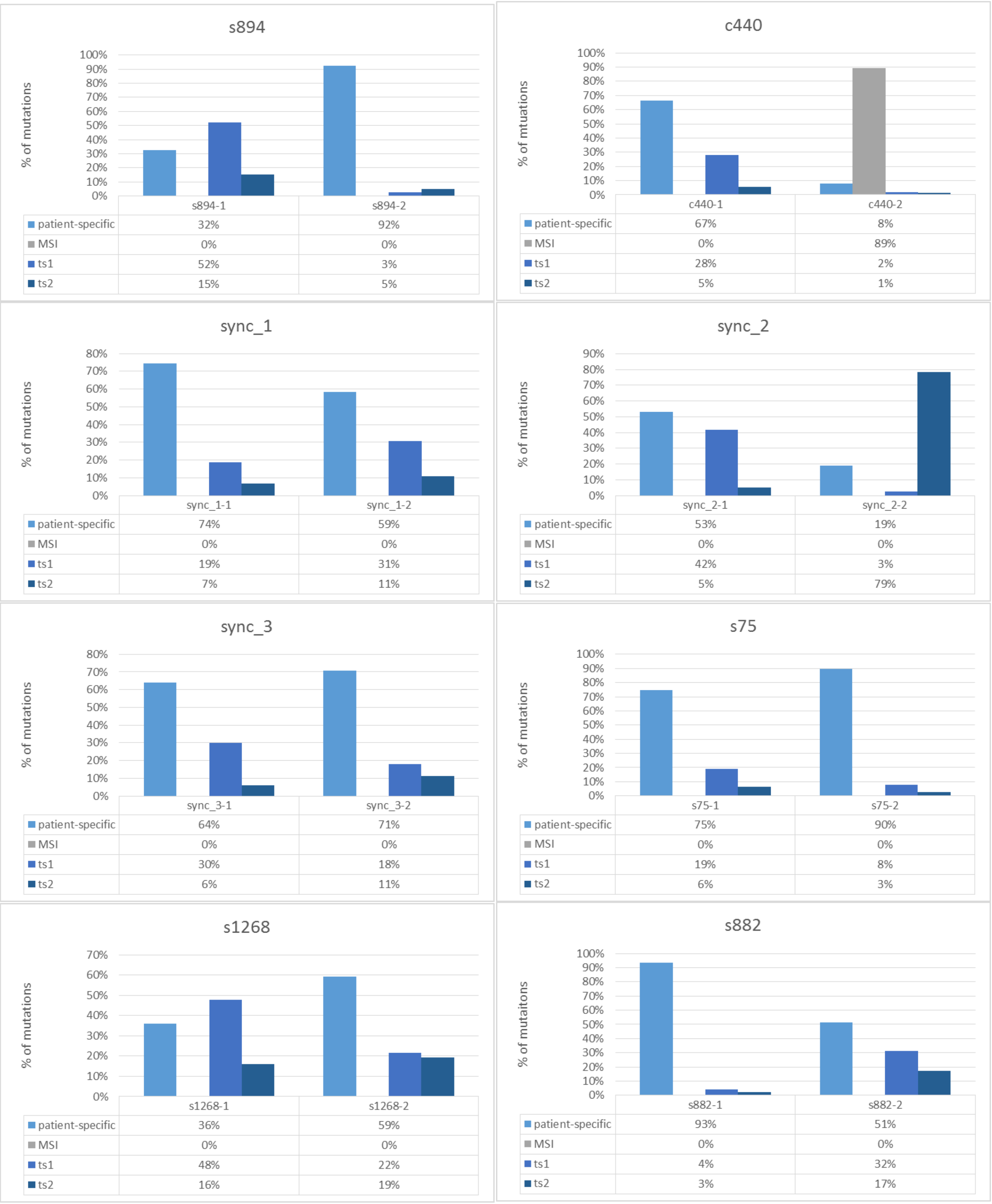

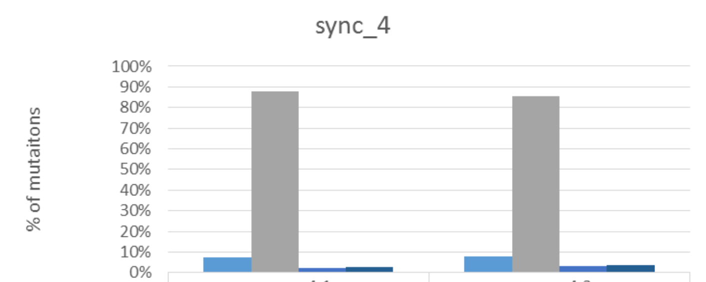


| | sync_4-1 | sync_4-2 |
|---|---|---|
| patient-specific | 7% | 8% |
| MSI | 88% | 86% |
| ts1 | 2% | 3% |
| ts2 | 3% | 4% |

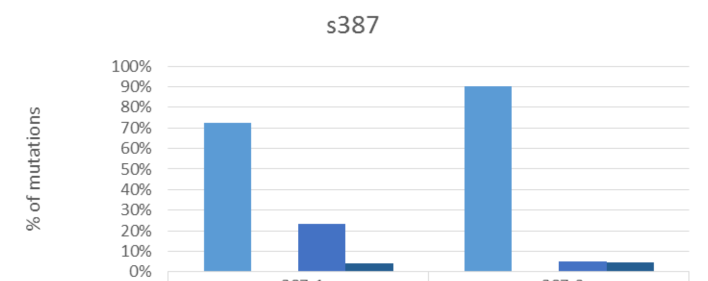


| | s387-1 | s387-2 |
|---|---|---|
| patient-specific | 73% | 90% |
| MSI | 0% | 0% |
| ts1 | 23% | 5% |
| ts2 | 4% | 5% |

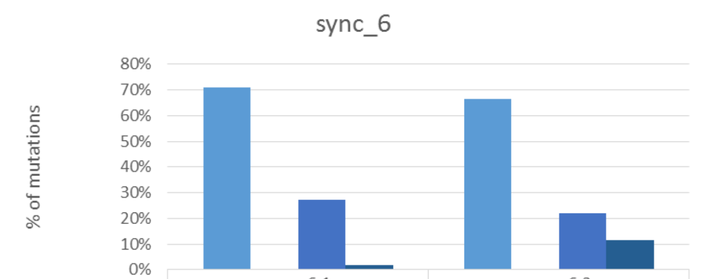


| | sync_6-1 | sync_6-2 |
|---|---|---|
| patient-specific | 71% | 67% |
| MSI | 0% | 0% |
| ts1 | 27% | 22% |
| ts2 | 2% | 11% |

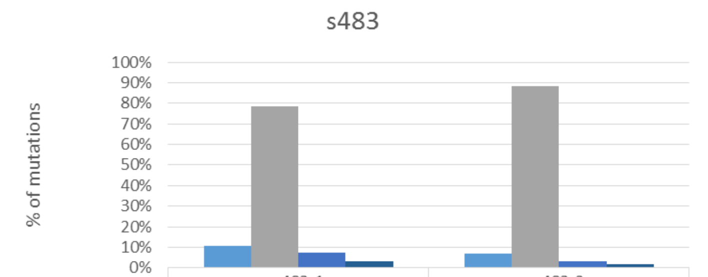


| | s483-1 | s483-2 |
|---|---|---|
| patient-specific | 11% | 7% |
| MSI | 78% | 88% |
| ts1 | 7% | 3% |
| ts2 | 3% | 2% |

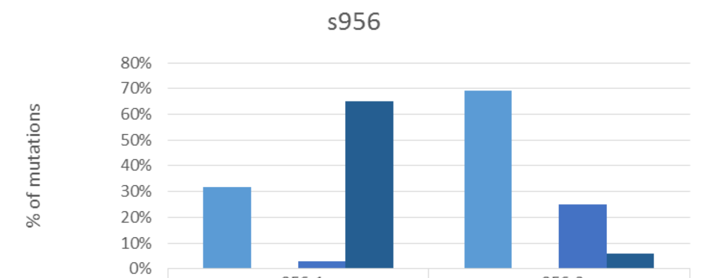


| | s956-1 | s956-2 |
|---|---|---|
| patient-specific | 32% | 69% |
| MSI | 0% | 0% |
| ts1 | 3% | 25% |
| ts2 | 65% | 6% |

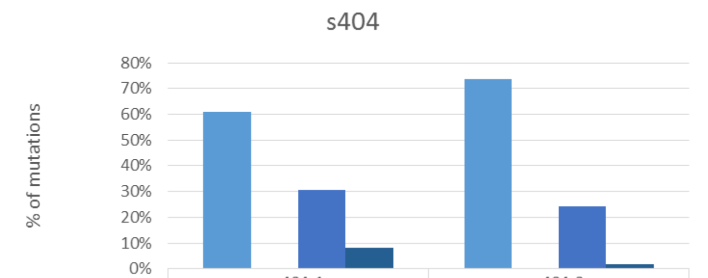


| | s404-1 | s404-2 |
|---|---|---|
| patient-specific | 61% | 74% |
| MSI | 0% | 0% |
| ts1 | 31% | 24% |
| ts2 | 8% | 2% |

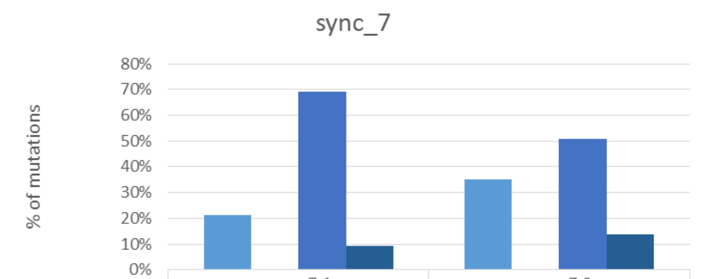


| | sync_7-1 | sync_7-2 |
|---|---|---|
| patient-specific | 21% | 35% |
| MSI | 0% | 0% |
| ts1 | 69% | 51% |
| ts2 | 9% | 14% |

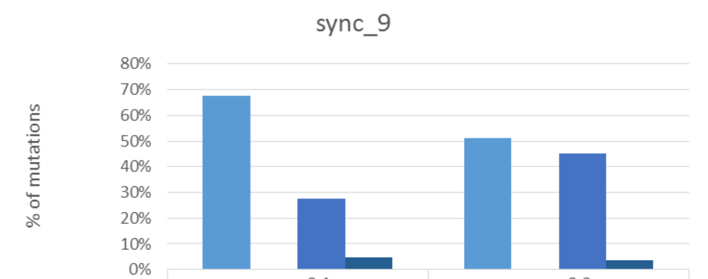


| | sync_9-1 | sync_9-2 |
|---|---|---|
| patient-specific | 68% | 51% |
| MSI | 0% | 0% |
| ts1 | 28% | 45% |
| ts2 | 5% | 4% |

## sync_10

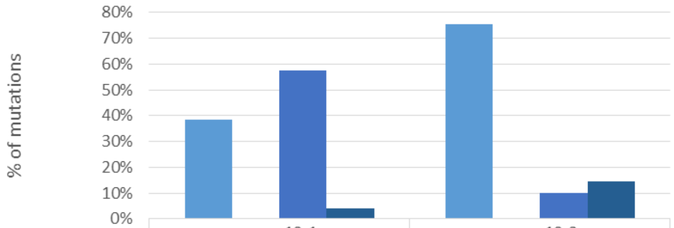


| | sync_10-1 | sync_10-2 |
|---|---|---|
| patient-specific | 38% | 76% |
| MSI | 0% | 0% |
| ts1 | 58% | 10% |
| ts2 | 4% | 14% |

## sync_11

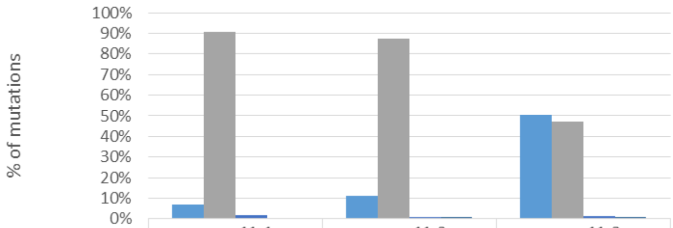


| | sync_11-1 | sync_11-2 | sync_11-3 |
|---|---|---|---|
| patient-specific | 7% | 11% | 51% |
| MSI | 91% | 87% | 47% |
| ts1 | 2% | 1% | 1% |
| ts2 | 1% | 1% | 1% |

## s1283

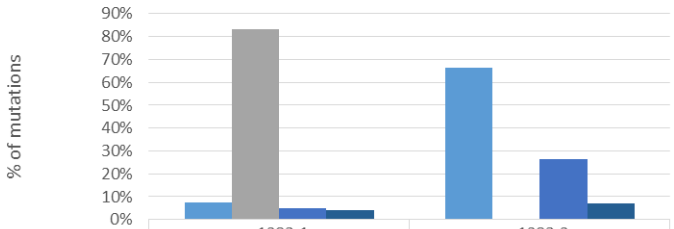


| | s1283-1 | s1283-2 |
|---|---|---|
| patient-specific | 8% | 67% |
| MSI | 83% | 0% |
| ts1 | 5% | 26% |
| ts2 | 4% | 7% |

## s1036

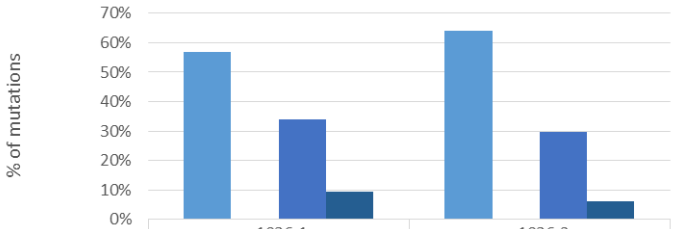


| | s1036-1 | s1036-2 |
|---|---|---|
| patient-specific | 57% | 64% |
| MSI | 0% | 0% |
| ts1 | 34% | 30% |
| ts2 | 9% | 6% |

## c110

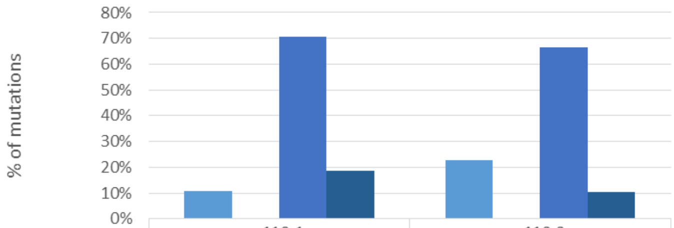


| | c110-1 | c110-2 |
|---|---|---|
| patient-specific | 11% | 23% |
| MSI | 0% | 0% |
| ts1 | 71% | 67% |
| ts2 | 18% | 10% |

## c117

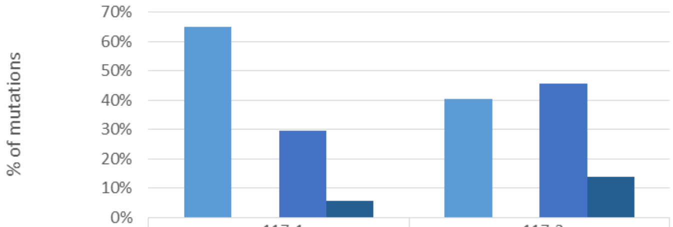


| | c117-1 | c117-2 |
|---|---|---|
| patient-specific | 65% | 40% |
| MSI | 0% | 0% |
| ts1 | 30% | 46% |
| ts2 | 6% | 14% |

## s934

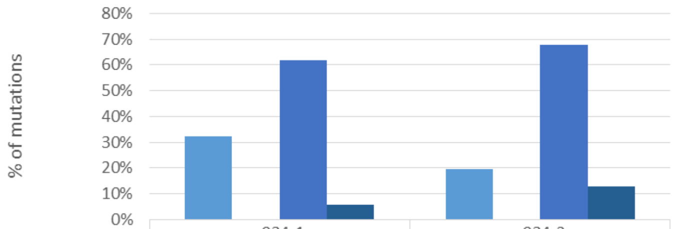


| | s934-1 | s934-2 |
|---|---|---|
| patient-specific | 32% | 19% |
| MSI | 0% | 0% |
| ts1 | 62% | 68% |
| ts2 | 6% | 13% |
