# Supplementary material for: Exome and immune cell score analyses reveal great variation within synchronous primary colorectal cancers
Source: Br J Cancer. 2019 Mar 21;120(9):922–30. doi: 10.1038/s41416-019-0427-4 (PMC6734647; doi:10.1038/s41416-019-0427-4)
Supplement: Supplementary file 6 — Supplementary Table 5. The altered known cancer pathways in synchronous cancers [file 41416_2019_427_MOESM6_ESM.pdf]

Supplementary Table 5. The altered known cancer pathways in synchronous cancers. Number refers to the number of genes with a nonsynonymous mutation related to each pathway. The intensity of the color red reflects the number of genes mutated within each pathway in the tumor. Shared pathways (and %) column refers to the number of shared mutated pathways within the tumor pair.

	ATM	p53	Wnt/β-catenin	ErbB	PTEN	PI3K/AKT	ERK/MAPK	TGF-β	shared pathways	%
**c110-1**	2	1	1			1	1		5/8	63 %
**c110-2**	1	1	2			1	1	2		
**c117-1**	2	4	2	4	4	4	4	2	7/8	88 %
**c117-2**	1	2	2	2	2	2	2			
**c440-1**		1	2					1	3/8	38 %
**c440-2**	13	12	19	9	9	9	13	7		
**s75-1**	2	3	3	5	4	5	8	2	7/8	88 %
**s75-2**		1	3	2	4	3	3	2		
**s387-1**	1	1	2	2	2	3	2	2	5/8	63 %
**s387-2**	1	1	1		1	1				
**s404-1**	1		1	2	2	1	2	3	7/8	88 %
**s404-2**	1	2	4	1	2	2	3	1		
**s483-1**	7	3	6	2	6	4	6	5	8/8	100 %
**s483-2**	10	7	14	6	9	11	14	12		
**s882-1**	1	1	2		1	2			5/8	63 %
**s882-2**	3	3	3	2	2	2	3	1		
**s894-1**	1	2	2	3	4	2	4	1	1/8	13 %
**s894-2**								1		
**s934-1**	1		2	1	3	3	2	1	4/8	50 %
**s934-2**	1	3	5		1	2				
**s956-1**	1	2	5	2	3	3	3	1	7/8	88 %
**s956-2**		2	1	3	4	4	2	2		
**s1036-1**		2	2	2	1	1	1	1	3/8	38 %
**s1036-2**			3	2	1					
**s1268-1**	2	2	1			2	1		5/8	63 %
**s1268-2**	1	2	3	3	1	2	3	1		
**s1283-1**	8	11	14	9	6	7	8	3	8/8	100 %
**s1283-2**	2	1	2	1	3	2	1	2		
**sync_1-1**	2	1	3	2	3	4	3	1	8/8	100 %
**sync_1-2**	3	2	4	3	1	2	3	2		
**sync_2-1**	2	1	3	3	2	2	2	3	8/8	100 %
**sync_2-2**	2	1	5	1	1	5	2	2		
**sync_3-1**	2	1	3			1	1	1	4/8	50 %
**sync_3-2**			1	2	1	1	1	2		
**sync_4-1**	11	16	23	19	19	16	21	15	8/8	100 %
**sync_4-2**	11	11	13	11	14	13	17	7		
**sync_6-1**	1	2	2	4	3	3	3	1	8/8	100 %
**sync_6-2**	1	1	3	1	1	2	1	1		
**sync_7-1**			4	1	1	1	1	1	6/8	75 %
**sync_7-2**	2	2	3	1	1	2	1	1		
**sync_9-1**		1	1						2/8	25 %
**sync_9-2**	2	4	2	1	1	2	1			
**sync_10-1**	1	1	3	2	2	3	2	3	7/8	88 %
**sync_10-2**	2	1	3	1	2	2	1			
**sync_11-1**	8	14	18	6	9	7	12	9		
**sync_11-2**	1	5	10	10	6	4	10	8	5/8	63 %
**sync_11-3**		2	4	1		1		1		
